# Supplementary material for: Epidemiological data of an influenza A/H5N1 outbreak in elephant seals in Argentina indicates mammal-to-mammal transmission
Source: Nat Commun. 2024 Nov 11;15:9516. doi: 10.1038/s41467-024-53766-5 (PMC11555070; doi:10.1038/s41467-024-53766-5)

Supplementary Table 1. Summary of southern elephant seal haul-out or stranding events recorded along the western coast of Golfo Nuevo, 2022–2023.

Event	Date	Latitude	Longitude	Age class/sex	Remarks
1	23-Sep-2022	-42.77134	-65.02702	Unknown	Living. Returned to the water before a more thorough inspection was possible.
2	27-Oct-2022	-42.77361	-65.02484	Weaner	Living. Apparently healthy and in good body condition. Translocated to a quieter beach.
3	6-Nov-2022	-42.81775	-64.89000	Weaner	Living. Apparently healthy but with an injury on the lower back. Significantly larger than the individual from event 2.
4A	20-Oct-2023	-42.59293	-64.81035	Subadult male	Living. Returned to the water before a more thorough inspection was possible.
4B	22-Oct-2023	-42.64036	-64.96321	Subadult male	Dead. From the size and general appearance, it is believed to be the same individual as event 4A. Good body condition, with only minor injuries (round cuts to rear flippers).
5	24-Oct-2023	-42.76766	-65.03123	Adult female	Dead. Found early in the morning and buried immediately by municipal authorities before post-mortem examination was possible.
6	25-Oct-2023	-42.64035	-64.96330	Juvenile female	Living. Apparently healthy and in good body condition.
7	30-Oct-2023	-42.59714	-64.81811	Subadult male	Living. This animal was c. 4 year-old (subadult category 1) and was first seen on 30-Oct-2023 at 15:45h, presenting with lethargy, tremors and laboured breathing. These clinical signs persisted during the following two days. Yellowish nasal discharge was first noticed on 1-Nov-2023 at 12:00h, and the animal was found dead at 17:00h. When the carcass was sampled at 19:00h, blood-stained nasal discharge was also noticed. While accessing the thoracic cavity for lung swab collection, it was noticed that it was uncomfortably warm to touch (estimated c. 45°C), suggesting ante-mortem hyperthermia (maximum air temperature that day was 18.6°C at 13:30h).
8	16-Nov-2023	-42.78189	-65.00144	Juvenile female	Living. Returned to the water before a more thorough inspection was possible.
9	2-Dec-2023	-42.78397	-65.00962	Juvenile male	Living. Poor body condition but apparently healthy, about to initiate moult.

Supplementary Table 2. Quantification cycle (Cq) values for RT-qPCR targeting the matrix gene of influenza A viruses (IAV) or the hemagglutinin gene of strains from the H5 clade 2.3.4.4b. Asterisks indicate samples sequenced in this study.

Host (sample identification)	Collection date	Location	Sample	IAV	H5 clade 2.3.4.4b
Elephant seal pups (pooled samples from 4 individuals: CH-PD027, CH-PD032, CH-PD033, and CH-PD035)	10-Oct-2023	Punta Delgada	Brain	15.01	17.68
			Lung	21.77	23.98
			Tracheal	21.78	24.68
			Oronasal	25.28	27.65
			Rectal	24.59	28.99
South American terns (pooled samples from 5 individuals: CH-PD028, CH-PD029, CH-PD030, CH-PD031, and CH-PD038)	10-Oct-2023	Punta Delgada	Brain	15.25	Not determined
			Lung	20.31	Not determined
			Orotracheal	21.36	Not determined
			Cloacal	24.84	Not determined
Royal terns (pooled samples from 2 individuals: CH-PD034 and CH-PD036)	10-Oct-2023	Punta Delgada	Brain	19.55	Not determined
			Lung	22.48	Not determined
			Orotracheal	28.79	Not determined
			Cloacal	30.76	Not determined
South American terns (CH-PD037)	10-Oct-2023	Punta Delgada	Brain + Cloacal + Lung + Orotracheal*	15.65	Not determined
Elephant seal subadult (CH-PD053)	01-Nov-2023	Puerto Madryn	Lung + Oronasal + Rectal	29.45	31.39
			Rectal*	24.52	Not determined
			Oronasal	32.85	Not determined
			Lung	35.19	Not determined
Elephant seal pup (CH-PD027)	10-Oct-2023	Punta Delgada	Rectal*	30.25	Not determined
Elephant seal pup (CH-PD032)	10-Oct-2023	Punta Delgada	Brain*	17.78	Not determined
			Lung*	22.42	Not determined
			Rectal*	24.44	Not determined
			Tracheal*	25.95	Not determined
			Oronasal*	27.44	Not determined
Elephant seal pup (CH-PD035)	10-Oct-2023	Punta Delgada	Brain*	15.50	Not determined
South American tern (CH-PD030)	10-Oct-2023	Punta Delgada	Brain*	15.17	Not determined
Royal tern (CH-PD036)	10-Oct-2023	Punta Delgada	Brain*	19.91	Not determined

Supplementary Table 3. Mutation analysis of H5N1 HPAI viruses from wildlife sampled at Península Valdés, Argentina, October 2023. Here we show 64 variable sites relative to the reference strain A/goose/Guangdong/1/1996 (Gs/Gd). The first H5N1 HPAI virus detected in Argentina from a goose in February 2023 (α), H5N1 HPAI viruses from poultry between February and June 2023 (β), and H5N1 HPAI viruses from sea lions in August 2023 (δ) are included for comparison. Mutations previously reported to be associated with specific pathogenic phenotypes of interest are indicated by an asterisk (*). Sixteen mutations that also differ from H5N1 HPAI clade 2.3.4.4b (genotype B3.2) strains from North America and from goose/poultry strains from Argentina are highlighted in bold. These mutations are of particular interest because they occur more than once: 15 mutations are shared by strains recovered from marine mammals and seabirds in South America (highlighted in orange) and one mutation is exclusive to strains recovered from outbreaks in marine mammals and seabirds in Argentina (highlighted in red). HA mutations refer to H5 numbering.

Segment	Mutation	References		Other HPAI H5N1 from Argentina			This study							Remarks
		Gs/Gd	B3.2 (N. Am.)	Goose Argentina[α]	Poultry Argentina[β]	Sea lions Argentina[δ]	Elephant seal				South American tern		Royal tern	
							CH-PD027	CH-PD032	CH-PD035	CH-PM053	CH-PD030	CH-PD037	CH-PD036	
PB2	**V122I**	V	V	V	V	V/I	V	V	V	I	V	V	I	Present in marine mammals in Brazil and Uruguay.
	I463V	I	V	V	V	V	V	V	V	V	V	V	V	Present in most genomes in South America.
	L464M	L	M	M	M	M	M	M	M	M	M	M	M	Present in most genomes in South America.
	V478I	V	I	I	I	I	I	I	I	I	I	I	I	Present in most genomes in South America.
	**Q591K***	Q	Q	Q	Q	K	K	K	K	K	K	K	K	Present in marine mammals in Peru, Chile, Brazil and Uruguay. Present in human case in Chile.
	I616V	I	I/V	V	V	V	V	V	V	V	V	V	V	Present in most genomes in South America.
	E627K*	E	E	E	E	E	E	E	E	E	E	E	E	Not present in H5N1 strains from South America.
	**D701N***	D	D	D	D	N	N	N	N	N	N	N	N	Present in marine mammals in Peru, Chile, Brazil and Uruguay. Present in human case in Chile.
PB1	T59S	T	T/S	S	S	S	S	S	S	S	S	S	S	Present in all genomes in South America.
	E264D	E	E/D	D	D	D	D	D	D	D	D	D	D	Present in most genomes in South America.
	L378M	L	L	M	M	M	M	M	M	M	M	M	M	Present in most genomes in South America.
	G399D	G	D	D	D	D	D	D	D	D	D	D	D	Present in most genomes in South America.
	K429R	K	K/R	R	R	R	R	R	R	R	R	R	R	Present in most genomes in South America.
	**S515A**	S	S	S	S	A	A	A	A	A	A	A	A	Present in marine mammals in Peru, Chile, Brazil and Uruguay.

Segment	Mutation	References		Other HPAI H5N1 from Argentina			This study							Remarks
		Gs/Gd	B3.2 (N. Am.)	Goose Argentina[α]	Poultry Argentina[β]	Sea lions Argentina[δ]	Elephant seal				South American tern		Royal tern	
							CH-PD027	CH-PD032	CH-PD035	CH-PM053	CH-PD030	CH-PD037	CH-PD036	
	**L548F**	L	L	L	L	L/F	F	F	F	L	F	L	L	Present in marine mammals in Peru and Chile (NOT in marine mammals in Brazil and Uruguay).
	**Q621K**	Q	Q	Q	Q	Q/K	K	K	K	Q	K	K	Q	Present exclusively in multi-species outbreaks in coastal Argentina.
PB1-F2	T7I	T	T/I	I	I	I	I	I	I	I	I	I	I	Present in most genomes in South America.
	S12L	S	S/L	L	L	L	L	L	L	L	L	L	L	Present in most genomes in South America.
	N17S	N	N/S	S	S	S	S	S	S	S	S	S	S	Present in most genomes in South America.
	R21K	R	R/K	K	K	K	K	K	K	K	K	K	K	Present in most genomes in South America.
	Y42C	Y	Y/C	C	C	C	C	C	C	C	C	C	C	Present in most genomes in South America.
	R48Q	R	R/Q	Q	Q	Q	Q	Q	Q	Q	Q	Q	Q	Present in most genomes in South America.
	Q54R	Q	Q/R	R	R	R	R	R	R	R	R	R	R	Present in most genomes in South America.
	I55T	I	I/T	T	T	T	T	T	T	T	T	T	T	Present in most genomes in South America.
	Y57S	Y	Y/S	S	S	S	S	S	S	S	S	S	S	Present in most genomes in South America.
	W58L	W	W/L	L	L	L	L	L	L	L	L	L	L	Present in most genomes in South America.
	N66S*	N	S	S	S	S	S	S	S	S	S	S	S	Present in most genomes in South America.
	G70E	G	G/E	E	E	E	E	E	E	E	E	E	E	Present in most genomes in South America.
PA	**A20T**	A	A	A	A	T	T	T	T	T	T	T	T	Present in marine mammals in Peru, Chile, Brazil and Uruguay. Present in human case in Chile.
	**R57Q**	R	R	R	R	Q	Q	Q	Q	Q	Q	Q	Q	Present in marine mammals in Peru, Chile, Brazil and Uruguay. Present in human case in Chile.
	I61M*	I	I/M	M	M	M	M	M	M	M	M	M	M	Present in most genomes in South America. Altered endonuclease activity.

Segment	Mutation	References		Other HPAI H5N1 from Argentina			This study							Remarks
							Elephant seal				South American tern		Royal tern	
		Gs/Gd	B3.2 (N. Am.)	Goose Argentina[α]	Poultry Argentina[β]	Sea lions Argentina[δ]	CH-PD027	CH-PD032	CH-PD035	CH-PM053	CH-PD030	CH-PD037	CH-PD036	
	**M86I**	M	M	M	M	I	I	I	I	I	I	I	I	Present in marine mammals in Peru, Chile, Brazil and Uruguay. Present in human case in Chile.
	**E237A**	E	E	E	E	E	E	E	E	A	E	E	A	Present in marine mammals in Peru, Chile, Brazil and Uruguay.
	M441V	M	M/V	V	V	V	V	V	V	V	V	V	V	Present in most genomes in South America.
	**M548I**	M	M	M	M	I	I	I	I	I	I	I	I	Present in marine mammals in Peru, Chile, Brazil and Uruguay. Present in human case in Chile.
	T608S	T	T/S	S	S	S	S	S	S	S	S	S	S	Present in most genomes in South America.
HA (H5)	D94N*	D	S	S	S	S	S	S	S	S	S	S	S	Increased virus binding to α2–6.
	S121N*	S	S	S	S	S	S	S	S	S	S	S	S	Increased virus binding to α2–6.
	S133A*	S	A	A	A	S/A	S	S	S	A	A	S	A	Present in most genomes in South America. Increased pseudovirus binding to α2-6.
	S155N*	S	D	D	D	D	D	D	D	D	D	D	D	Increased virus binding to α2–6.
	D183G*	D	N	N	N	N	N	N	N	N	N	N	N	Increased virus binding to α2–6.
	V210I*	V	V/A	A	A	A	A	A	A	A	A	A	A	Increased virus binding to α2–6.
	K218E*	K	Q	Q	Q	Q	Q	Q	Q	Q	Q	Q	Q	Increased virus binding to α2-3 and α2-6.
	S223N*	S	R	R	R	R	R	R	R	R	R	R	R	Increased virus binding to α2-3 and α2-6.
NP	**I119T**	I	I	I	I	T	T	T	T	T	T	T	T	Present in marine mammals in Peru, Chile, Brazil and Uruguay. Present in human case in Chile.
	F230L	F	F/L	L	L	L	L	L	L	L	L	L	L	Present in all genomes in South America.
	S450N	S	S/N	N	N	N	N	N	N	N	N	N	N	Present in all genomes in South America.
NA	I8T	I	I/T	T	T	T	T	T	T	T	T	T	T	Present in all genomes in South America.
	A81T	A	T	T	T	T	T	T	T	T	T	T	T	Present in all genomes in South America.
	L269M	L	L/M	M	M	M	M	M	M	M	M	M	M	Present in most genomes in South America.
	S339P	S	S/P	P	P	P	P	P	P	P	P	P	P	Present in most genomes in South America.

Segment	Mutation	References		Other HPAI H5N1 from Argentina			This study							Remarks
		Gs/Gd	B3.2 (N. Am.)	Goose Argentina[α]	Poultry Argentina[β]	Sea lions Argentina[δ]	Elephant seal				South American tern		Royal tern	
							CH-PD027	CH-PD032	CH-PD035	CH-PM053	CH-PD030	CH-PD037	CH-PD036	
M1	N85S	N	N/S	S	S	S	S	S	S	S	S	S	S	Present in most genomes in South America.
	N87T	N	N	T	T	T	T	T	T	T	T	T	T	Present in most genomes in South America.
	K101R*	K	K/R	R	R	R	R	R	R	R	R	R	R	Present in all genomes in South America.
	A200V	A	A/V	V	V	V	V	V	V	V	V	V	V	Present in most genomes in South America.
M2	R61G	R	G	G	G	G	G	G	G	G	G	G	G	Present in all genomes in South America.
NS1	**L21R**	L	R	R	R	Q	Q	Q	Q	Q	Q	Q	Q	Present in marine mammals in Peru, Chile, Brazil and Uruguay. Present in human case in Chile.
	**D26E**	D	E	E	E	K	K	K	K	K	K	K	K	Present in marine mammals in Peru, Chile, Brazil and Uruguay. Present in human case in Chile.
	**D53G**	D	D	D	D	G	G	G	G	G	G	G	G	Present in marine mammals in Peru, Chile, Brazil and Uruguay. Present in human case in Chile.
	Y103F	Y/F	F	F	F	F	F	F	F	F	F	F	F	Present in all genomes in South America.
	M116S	M/C	S/C	S	S	S	S	S	S	S	S	S	S	Present in most genomes in South America.
	D139N	D	D/N	N	N	N	N	N	N	N	N	N	N	Present in most genomes in South America.
	A223E*	A	A/E	E	E	E	E	E	E	E	E	E	E	Present in most genomes in South America.
	**V226I**	V	V/I	I	I	T	T	T	T	T	T	T	T	V226T present in marine mammals in Peru, Chile, Brazil and Uruguay. Present in human case in Chile.

Supplementary Table 4. Mutations in H5N1 HPAI viruses from wildlife sampled in October 2023 at Península Valdés (asterisks) and from previous cases in wildlife in Argentina in August 2023 (crosses) compared with other representative H5N1 HPAI viruses from genotype B3.2 and the original Gs/Gd reference strain (A/goose/Guangdong/1/1996). The first detection of H5N1 HPAI virus in Argentina (from an Andean goose in February 2023) is shown in italics. Mutations known to be associated with increased virulence, transmission, or mammalian host adaptation are indicated with hash symbols. Dots represent amino acids identical to those present in the reference strain. HA mutations refer to H5 numbering.

			PB2			PB1			PA					HA	NP	NS1			
**Host group**	**Country**	**Host species**	**V122I**	**Q591K#**	**D701N#**	**S515A**	**L548F**	**Q621K**	**A20T**	**R57Q**	**M86I**	**E237A**	**M548I**	**S133A**	**I119T**	**L21R/Q**	**D26E/K**	**D53G**	**V226I/T**
Mammal	Argentina	Southern elephant seal*	V/I	K	N	A	L/F	Q/K	T	Q	I	E/A	I	S/A	T	Q	K	G	T
		South American sea lion†	V/I	K	N	A	L/F	Q/K	T	Q	I	·	I	S/A	T	Q	K	G	T
		South American fur seal†	·	K	N	A	F	K	T	Q	I	·	I	?	T	Q	K	G	T
	Brazil	South American sea lion	I	K	N	A	·	·	T	Q	I	A	I	A	T	Q	K	G	T
	Chile	Human	·	K	N	·	·	·	T	Q	I	·	I	A	T	Q	K	G	T
		Chilean dolphin	·	·	N	A	F	·	·	Q	·	·	·	A	T	R	K	G	T
		Burmeister's porpoise	·	Q/K	N	A	F	·	·	Q	M/I	·	M/I	A	T	R/Q	K	G	T
		South American sea lion	·	Q/K	N	A	F	·	·	Q	M/I	·	M/I	A	T	R/Q	K	G	I/T
	Peru	Common dolphin	·	·	·	·	·	·	·	·	·	·	·	A	·	R	E	·	I
		South American sea lion	·	Q/K	N	S/A	L/F	·	·	R/Q	M/I	·	M/I	A	I/T	R/Q	E/K	D/G	I/T
	Uruguay	South American sea lion	I	K	N	A	·	·	T	Q	I	A	I	A	T	Q	K	G	T
		South American fur seal	I	K	N	A	·	·	T	Q	I	A	I	A	T	Q	K	G	T
	USA	Harbor seal	·	·	·	·	·	·	·	·	·	·	·	A	·	R	E	D/G	I
	Europe	Harbor seal	·	·	·	·	·	·	·	·	·	·	·	A	·	R	D/E	·	I
Wild bird	Argentina	South American tern*	·	K	N	A	L/F	K	T	Q	I	·	I	S/A	T	Q	K	G	T
		South American tern†	·	K	N	A	F	K	T	Q	I	·	I	S	T	Q	K	G	T
		Royal tern*	I	K	N	A	·	·	T	Q	I	A	I	A	T	Q	K	G	T
		Andean goose	·	·	·	·	·	·	·	·	·	·	·	A	·	R	E	·	I
	Brazil	Cabot's tern	·	·	·	·	·	·	·	·	·	·	·	A	I/T	R	E	·	I

	Chile	Gull [1]	·	·	·	S/A	·	·	·	Q	·	·	·	A	I/T	R	E/K	D/G	I
		Peruvian pelican	·	·	·	S/A	·	·	·	Q	·	·	·	A	I/T	R	E/K	G	I
		Sanderling	·	·	N	A	·	·	·	Q	·	·	·	A	·	R	K	G	I/T
		Tern [2]	·	·	N	S/A	·	·	·	Q	·	·	·	A	·	R	K	G	I
	Ecuador	Frigatebird/booby [3]	·	·	·	·	·	·	·	·	·	·	·	A	·	R	E	·	I
	Falkland Is.	Southern fulmar	·	K	N	A	·	·	T	Q	I	·	I	A	T	Q	K	G	T
	Peru	Guanay cormorant	·	·	·	·	·	·	·	R/Q	·	·	·	A	·	R	E	D/G	I
		Gull/tern [4]	·	·	·	S/A	L/F	·	·	R/Q	·	·	·	A	·	R	E/K	D/G	I
		Peruvian pelican	·	·	·	·	·	·	·	R/Q	·	·	·	A	·	R	E/K	D/G	I
	Uruguay	Black-necked swan	I	·	·	·	·	·	·	·	·	·	·	A	·	R	E	·	I
	Venezuela	Brown pelican	·	·	·	·	·	·	·	Q	·	·	·	A	·	Q	E	·	I
	South Georgia	Brown skua	·	·	·	·	·	·	·	·	·	·	·	A	·	R	E	·	I
Poultry	Argentina	Chicken	·	·	·	·	·	·	·	·	·	·	·	A	·	R	E	·	I
	Chile		·	·	·	S/A	·	·	·	R/Q	·	·	·	A	·	R	E	D/G	I
	Colombia		·	·	·	·	·	·	·	·	·	·	·	A	·	R	E	·	I
	Ecuador		·	·	·	·	·	·	·	·	·	·	·	A	·	R	E	·	I
	Peru		·	·	·	S/A	·	·	·	R/Q	·	·	·	A	·	R	E/K	D/G	I
	Uruguay		·	·	·	·	·	·	·	·	·	·	·	A	·	R	E	·	I
	USA		·	·	·	·	·	·	·	·	·	·	·	A	·	R	E	D/G	I
Gs/Gd	China	Goose	V	Q	D	S	L	Q	A	R	M	E	M	S	I	L	D	D	I

Notes: [1] Belcher's gull (*Larus belcheri*), brown-hooded gull (*Larus maculipennis*), dolphin gull (*Larus scoresbii*), Franklin's gull (*Larus pipixcan*), grey gull (*Larus modestus*), and kelp gull (*Larus dominicanus*). [2] Elegant tern (*Thalasseus elegans*) and inca tern (*Larosterna inca*). [3] Blue-footed booby (*Sula neubouxii*) and magnificent frigatebird (*Fregata magnificens*). [4] Belcher's gull, kelp gull and inca tern.

Supplementary Figure 1. Behavior and interspecies interactions at Punta Delgada (Península Valdés, Argentina) during an outbreak of H5N1 HPAI. (A) Subadult male elephant seal resting alone amidst carcasses of elephant seal adult females and pups. (B) Small group of female elephant seals (and their pups) resting in close proximity to elephant seal carcasses; a South American tern carcass is also visible on the bottom right of the image. (C) Adult female elephant seal with pup reacting aggressively when approached by a subadult male sea lion. (D) Subadult male sea lion attempting to copulate with a living elephant seal pup (arrow). (E) Kelp gulls scavenging on carcasses of elephant seal pups. (F) South American tern standing next to a dead adult elephant seal, with other carcasses of elephant seal pups and South American terns visible in the background.

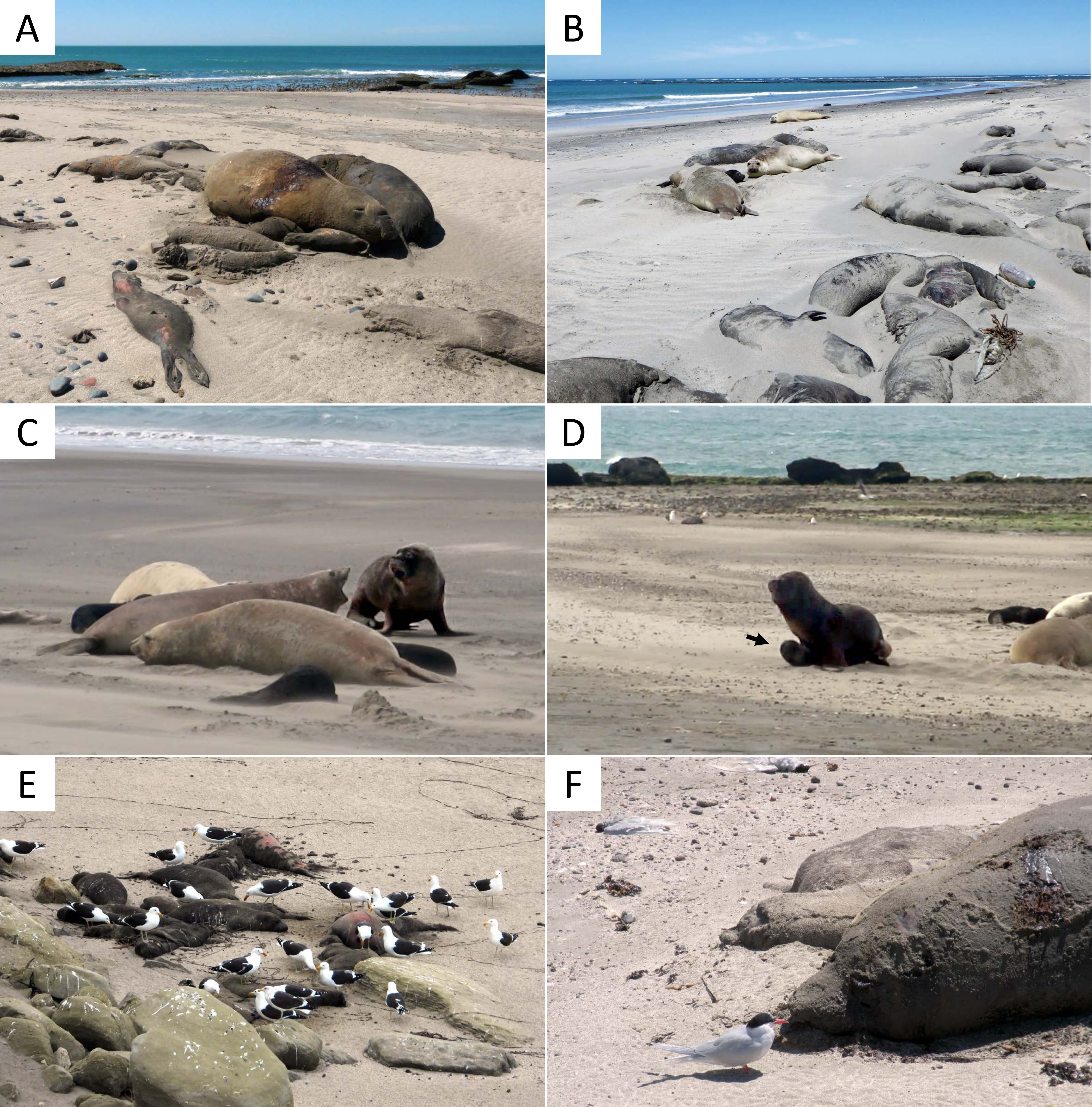
A
B
C
D
E
F

Supplementary Figure 2. Map of Península Valdés showing sites where samples were collected. (A) urban coast of Golfo Nuevo. (B) Punta Delgada elephant seal breeding colony. The overall breeding distribution of southern elephant seals was drawn from Ferrari et al. (2009). Elephant seal haul-outs in Golfo Nuevo are indicated using the same numbering as in Supplementary Table 1.

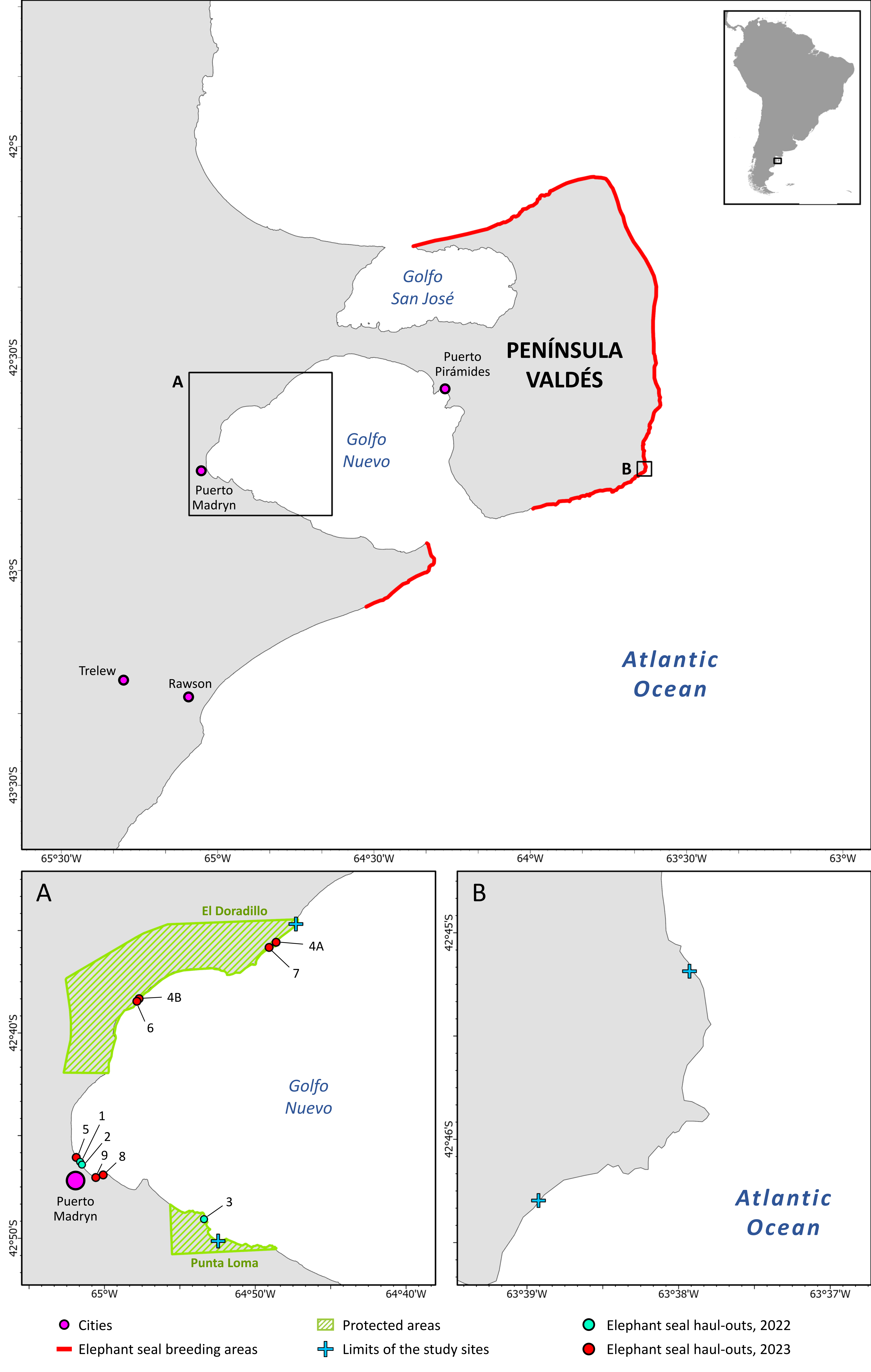
Golfo
San José
PENÍNSULA
VALDÉS
Puerto
Pirámides
A
Golfo
Nuevo
Puerto
Madryn
B
Trelew
Rawson
Atlantic
Ocean
42°S
42°30'S
43°S
43°30'S
65°30'W
65°W
64°30'W
64°W
63°30'W
63°W
A
El Doradillo
4A
7
4B
6
Golfo
Nuevo
1
5
2
9
8
Puerto
Madryn
3
Punta Loma
42°40'S
42°50'S
65°W
64°50'W
64°40'W
B
42°45'S
42°46'S
Atlantic
Ocean
63°39'W
63°38'W
63°37'W
Cities
Elephant seal breeding areas
Protected areas
Limits of the study sites
Elephant seal haul-outs, 2022
Elephant seal haul-outs, 2023

Supplementary Figure 3. Maximum likelihood tree inferred for the HA segment of 1,128 H5N1 2.3.4.4b viruses collected from South America, North America, and Eurasia during 2021-2023. The clade containing viruses from Argentina, Brazil, Chile, Falkland/Malvinas Islands, Peru, South Georgia and Uruguay is highlighted in red. Bootstrap values provided for all nodes. All branch lengths drawn to scale.

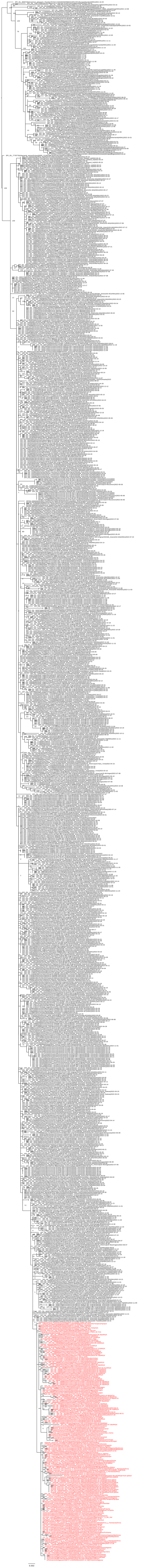

Supplementary Figure 4. Time-scale MCC tree. Same tree as Figure 2A, but with tip labels and bootstrap values. XML file and tree file available in Zenodo [https://doi.org/10.5281/zenodo.13923371].

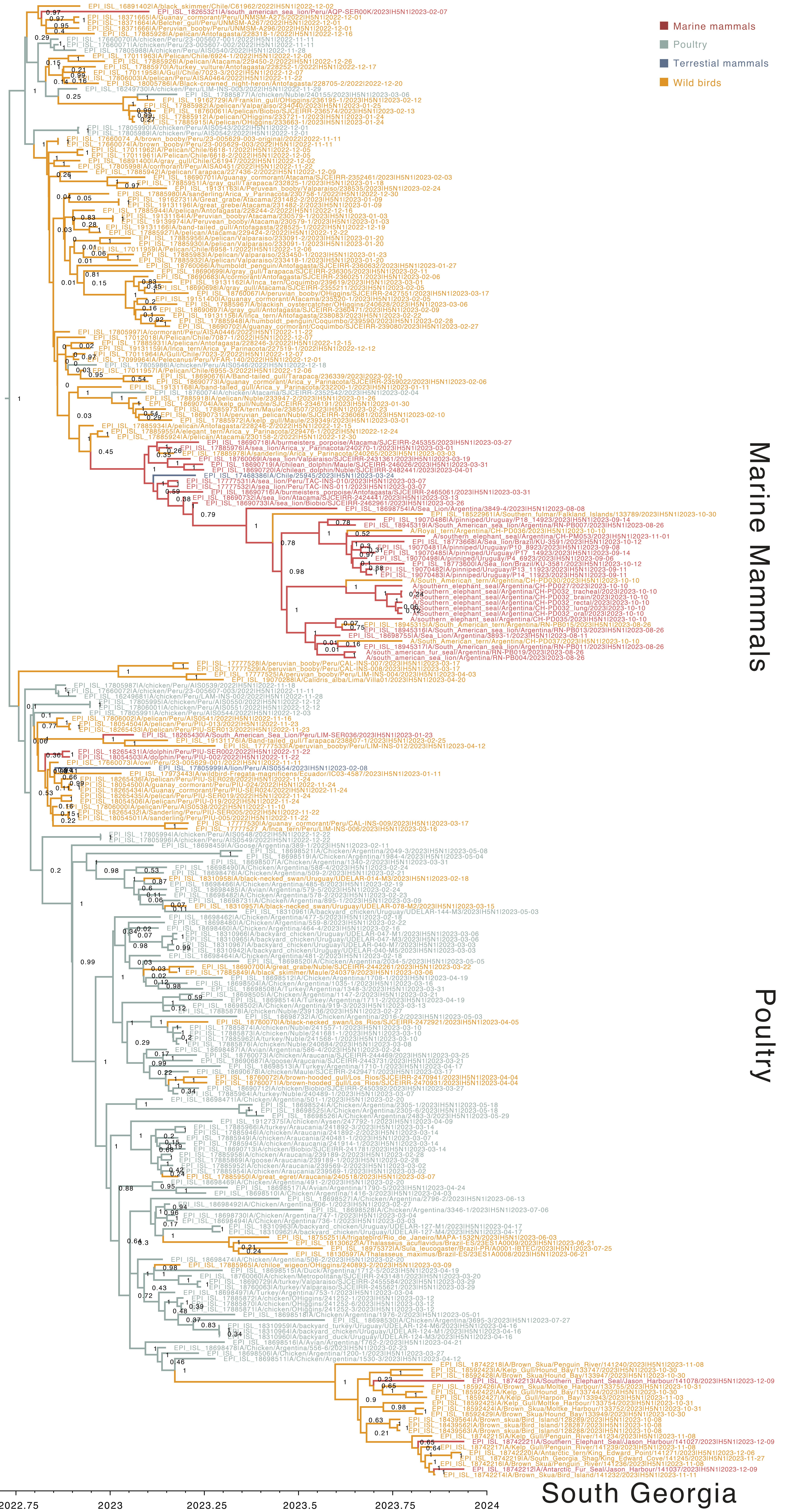

Marine mammals
Poultry
Terrestial mammals
Wild birds
Marine Mammals
Poultry
South Georgia
2022.75
2023
2023.25
2023.5
2023.75
2024

Supplementary Figure 5. Estimated rates of virus gene flow between location and hosts. Similar to Figure 2B, except with 95% HPD (highest posterior density) values.

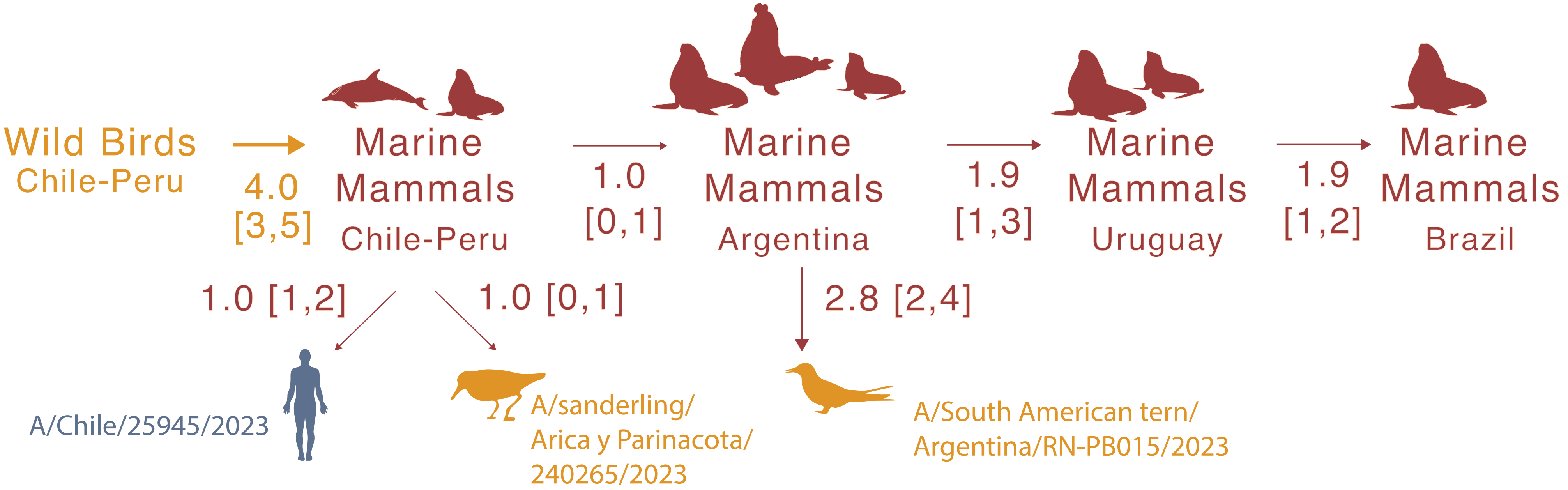
Wild Birds
Chile-Peru
4.0
[3,5]
Marine
Mammals
Chile-Peru
1.0
[0,1]
Marine
Mammals
Argentina
1.9
[1,3]
Marine
Mammals
Uruguay
1.9
[1,2]
Marine
Mammals
Brazil
1.0 [1,2]
1.0 [0,1]
2.8 [2,4]
A/Chile/25945/2023
A/sanderling/
Arica y Parinacota/
240265/2023
A/South American tern/
Argentina/RN-PB015/2023

Supplementary Figure 6. Time-scale MCC tree inferred using additional location states. Similar to Figure 2A and Supplementary Figure 4, but using the additional location state categories (n = 14) used in the phylogeographic Markov jump analysis presented in Figure 2B and Supplementary Figure 5. XML file and tree file available in Zenodo [https://doi.org/10.5281/zenodo.13923371].

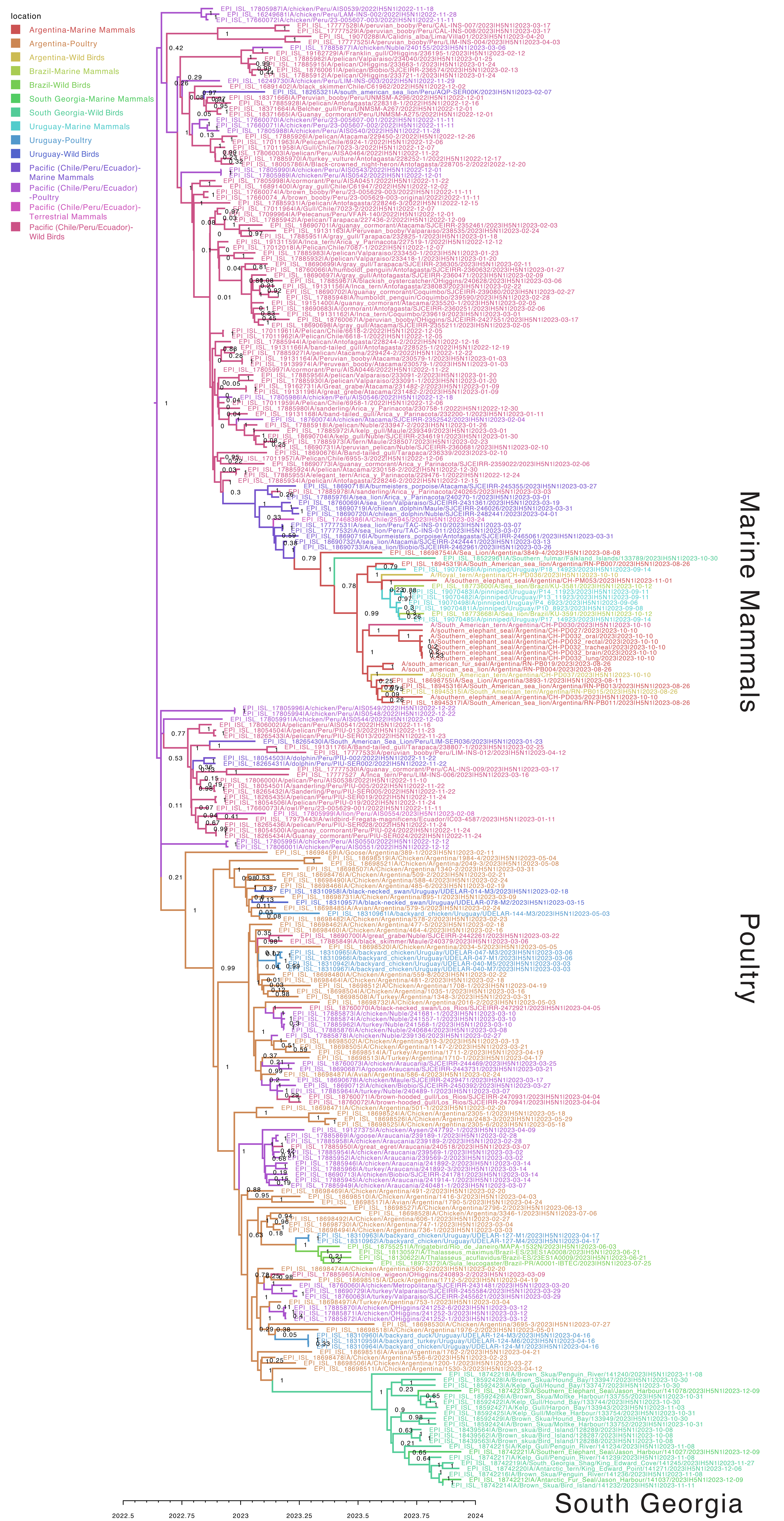
location
Argentina-Marine Mammals
Argentina-Poultry
Argentina-Wild Birds
Brazil-Marine Mammals
Brazil-Wild Birds
South Georgia-Marine Mammals
South Georgia-Wild Birds
Uruguay-Marine Mammals
Uruguay-Poultry
Uruguay-Wild Birds
Pacific (Chile/Peru/Ecuador)-Marine Mammals
Pacific (Chile/Peru/Ecuador)-Poultry
Pacific (Chile/Peru/Ecuador)-Terrestrial Mammals
Pacific (Chile/Peru/Ecuador)-Wild Birds
Marine Mammals
Poultry
South Georgia
2022.5
2022.75
2023
2023.25
2023.5
2023.75
2024

Supplementary Figure 7. Time-scale MCC tree inferred using a host-specific local clock (HSLC). Similar tree as Figure 2A in Zenodo [https://doi.org/10.5281/zenodo.13923371].

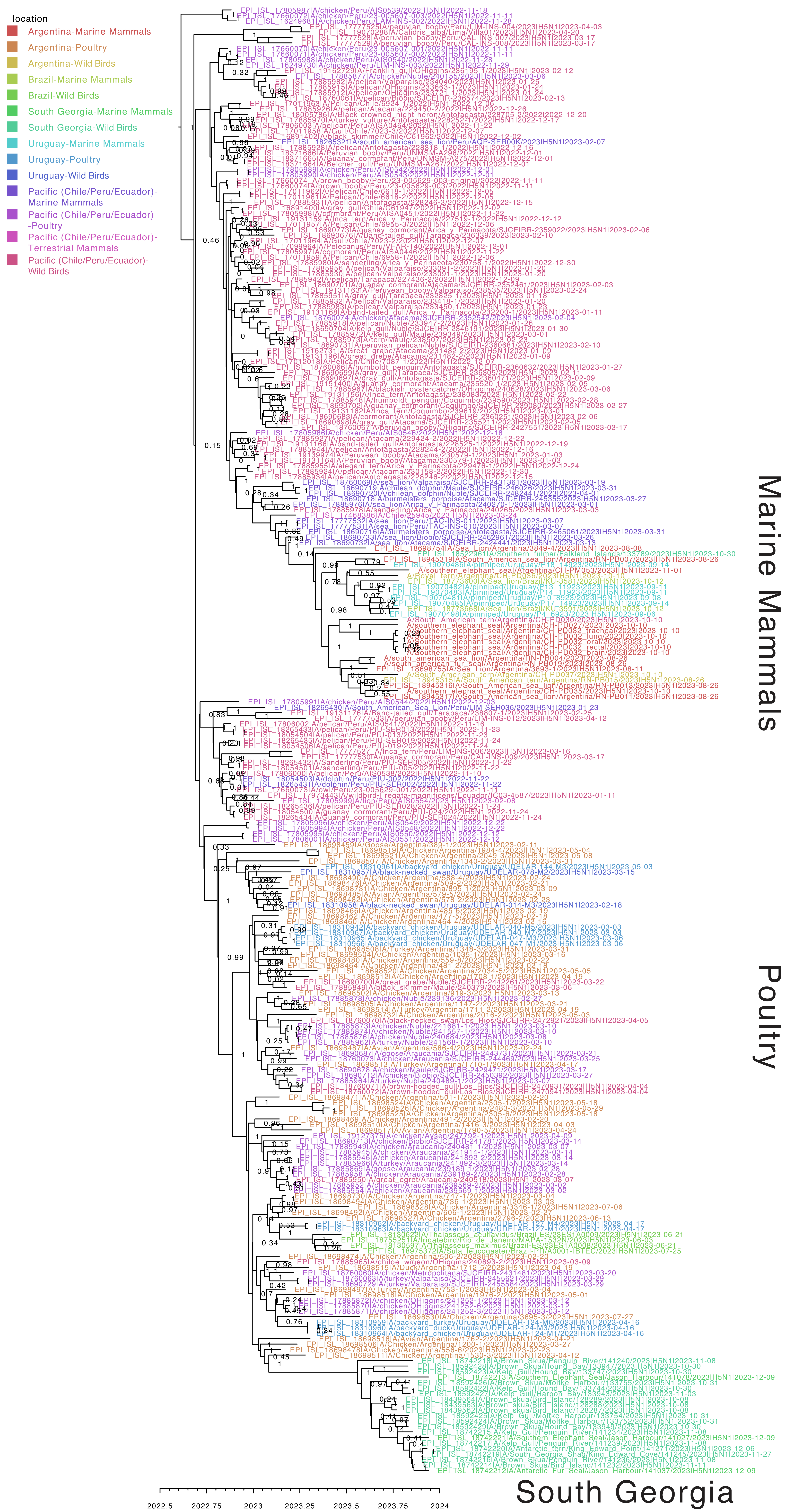

location
Argentina-Marine Mammals
Argentina-Poultry
Argentina-Wild Birds
Brazil-Marine Mammals
Brazil-Wild Birds
South Georgia-Marine Mammals
South Georgia-Wild Birds
Uruguay-Marine Mammals
Uruguay-Poultry
Uruguay-Wild Birds
Pacific (Chile/Peru/Ecuador)-Marine Mammals
Pacific (Chile/Peru/Ecuador)-Poultry
Pacific (Chile/Peru/Ecuador)-Terrestrial Mammals
Pacific (Chile/Peru/Ecuador)-Wild Birds
Marine Mammals
Poultry
South Georgia
2022.5
2022.75
2023
2023.25
2023.5
2023.75
2024

Supplementary Figure 8. Time-scale MCC tree inferred for third codon position only. Similar tree as Figure 2A, inferred for third codon position only. Shading similar to Supplementary Figure 5. XML file and tree file available in Zenodo [https://doi.org/10.5281/zenodo.13923371].

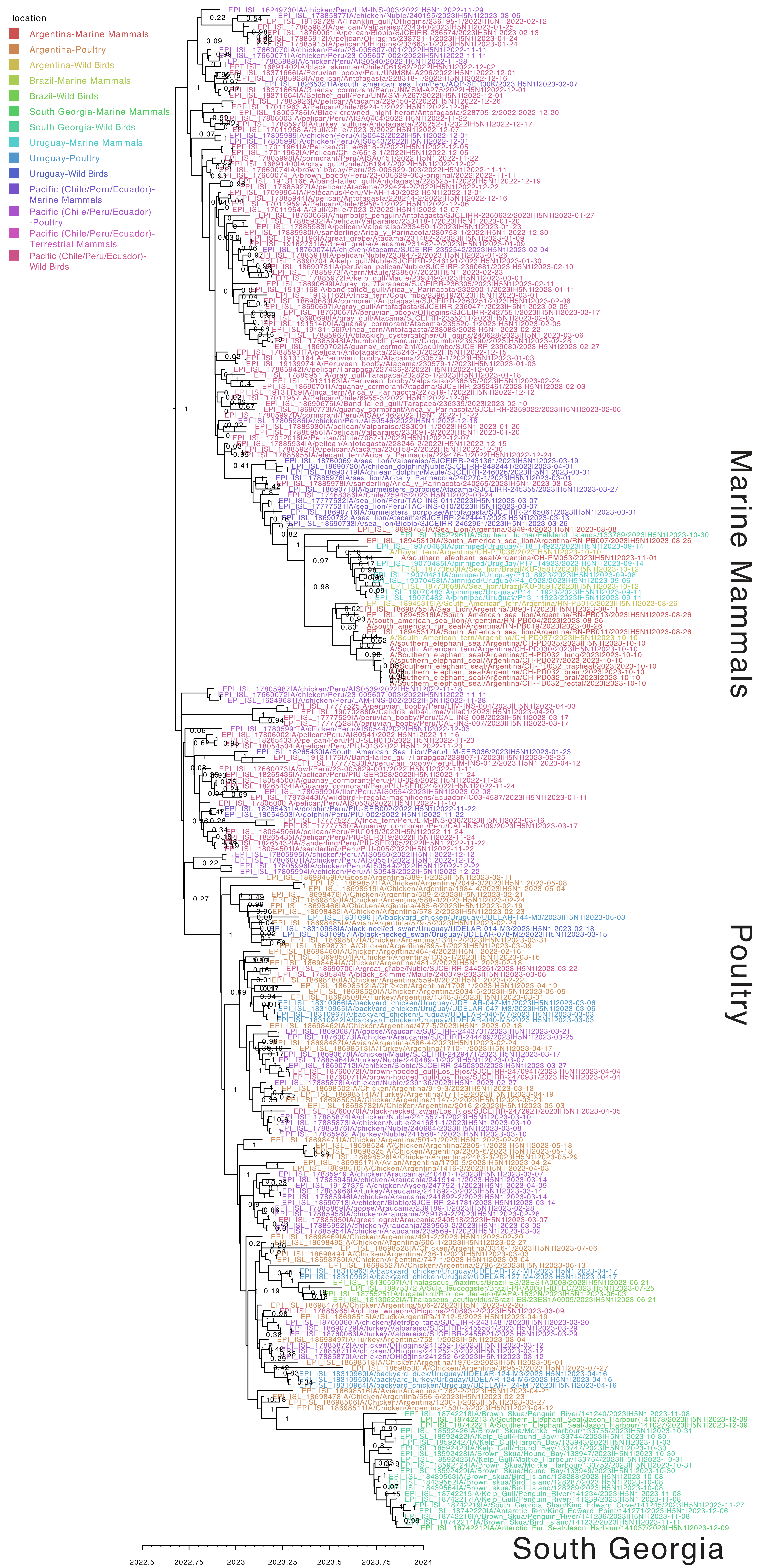
location
Argentina-Marine Mammals
Argentina-Poultry
Argentina-Wild Birds
Brazil-Marine Mammals
Brazil-Wild Birds
South Georgia-Marine Mammals
South Georgia-Wild Birds
Uruguay-Marine Mammals
Uruguay-Poultry
Uruguay-Wild Birds
Pacific (Chile/Peru/Ecuador)-Marine Mammals
Pacific (Chile/Peru/Ecuador)-Poultry
Pacific (Chile/Peru/Ecuador)-Terrestrial Mammals
Pacific (Chile/Peru/Ecuador)-Wild Birds
Marine Mammals
Poultry
South Georgia
2022.5
2022.75
2023
2023.25
2023.5
2023.75
2024

Supplementary Figure 9. Synonymous mutations. Synonymous (silent) mutations that arose in the marine mammal clade that are not observed in any other avian viruses included in this study from South America, mapped against the subsection of the MCC tree with the marine mammal clade (see Figure 2A). Virus names and associated mutations are colored by country. HA mutations refer to H5 numbering.

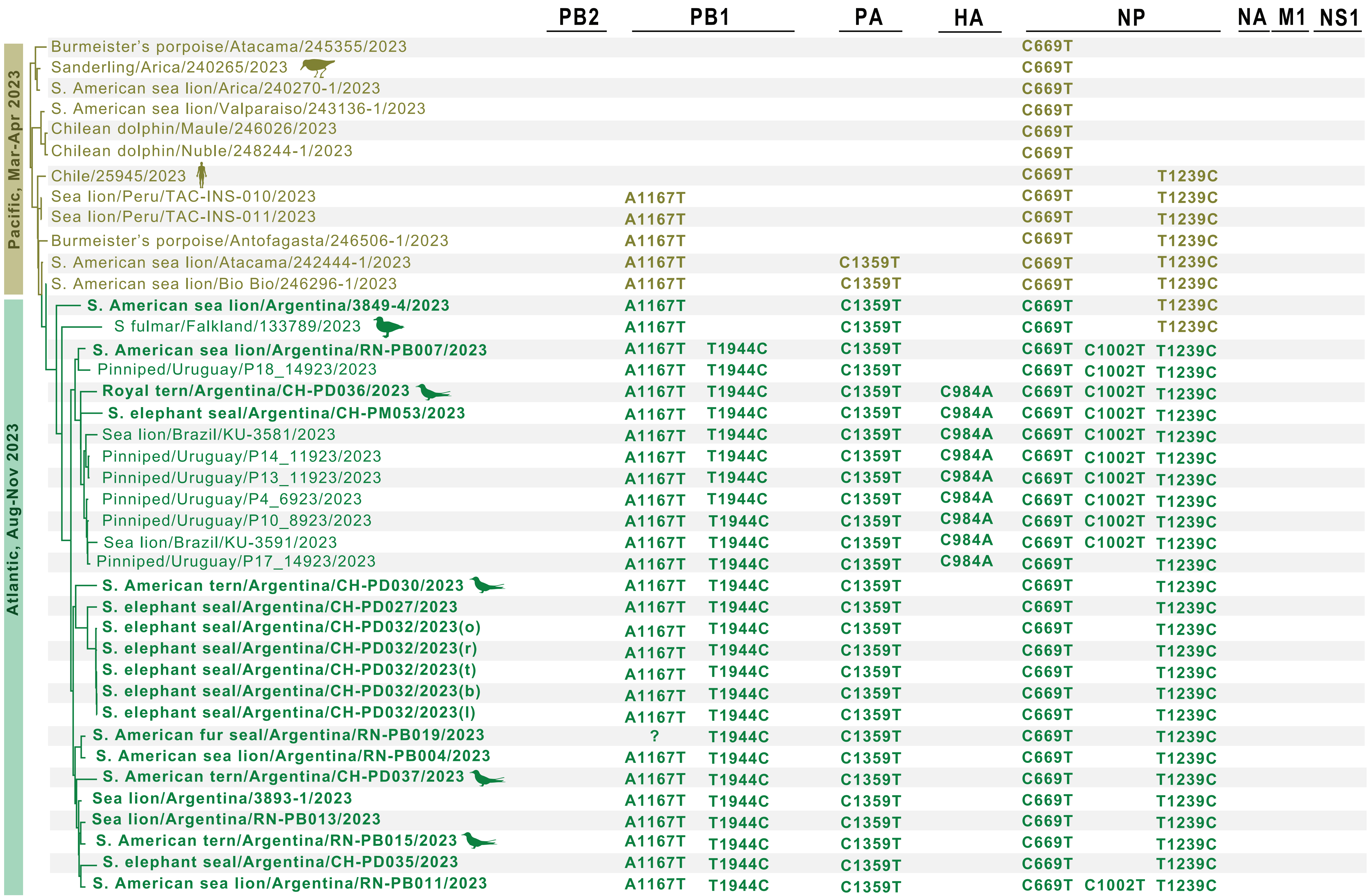
PB2
PB1
PA
HA
NP
NA
M1
NS1
Pacific, Mar-Apr 2023
Atlantic, Aug-Nov 2023
Burmeister’s porpoise/Atacama/245355/2023 C669T
Sanderling/Arica/240265/2023 C669T
S. American sea lion/Arica/240270-1/2023 C669T
S. American sea lion/Valparaiso/243136-1/2023 C669T
Chilean dolphin/Maule/246026/2023 C669T
Chilean dolphin/Nuble/248244-1/2023 C669T
Chile/25945/2023 C669T T1239C
Sea lion/Peru/TAC-INS-010/2023 A1167T C669T T1239C
Sea lion/Peru/TAC-INS-011/2023 A1167T C669T T1239C
Burmeister’s porpoise/Antofagasta/246506-1/2023 A1167T C669T T1239C
S. American sea lion/Atacama/242444-1/2023 A1167T C1359T C669T T1239C
S. American sea lion/Bio Bio/246296-1/2023 A1167T C1359T C669T T1239C
S. American sea lion/Argentina/3849-4/2023 A1167T C1359T C669T T1239C
S fulmar/Falkland/133789/2023 A1167T C1359T C669T T1239C
S. American sea lion/Argentina/RN-PB007/2023 A1167T T1944C C1359T C669T C1002T T1239C
Pinniped/Uruguay/P18_14923/2023 A1167T T1944C C1359T C669T C1002T T1239C
Royal tern/Argentina/CH-PD036/2023 A1167T T1944C C1359T C984A C669T C1002T T1239C
S. elephant seal/Argentina/CH-PM053/2023 A1167T T1944C C1359T C984A C669T C1002T T1239C
Sea lion/Brazil/KU-3581/2023 A1167T T1944C C1359T C984A C669T C1002T T1239C
Pinniped/Uruguay/P14_11923/2023 A1167T T1944C C1359T C984A C669T C1002T T1239C
Pinniped/Uruguay/P13_11923/2023 A1167T T1944C C1359T C984A C669T C1002T T1239C
Pinniped/Uruguay/P4_6923/2023 A1167T T1944C C1359T C984A C669T C1002T T1239C
Pinniped/Uruguay/P10_8923/2023 A1167T T1944C C1359T C984A C669T C1002T T1239C
Sea lion/Brazil/KU-3591/2023 A1167T T1944C C1359T C984A C669T C1002T T1239C
Pinniped/Uruguay/P17_14923/2023 A1167T T1944C C1359T C984A C669T T1239C
S. American tern/Argentina/CH-PD030/2023 A1167T T1944C C1359T C669T T1239C
S. elephant seal/Argentina/CH-PD027/2023 A1167T T1944C C1359T C669T T1239C
S. elephant seal/Argentina/CH-PD032/2023(o) A1167T T1944C C1359T C669T T1239C
S. elephant seal/Argentina/CH-PD032/2023(r) A1167T T1944C C1359T C669T T1239C
S. elephant seal/Argentina/CH-PD032/2023(t) A1167T T1944C C1359T C669T T1239C
S. elephant seal/Argentina/CH-PD032/2023(b) A1167T T1944C C1359T C669T T1239C
S. elephant seal/Argentina/CH-PD032/2023(l) A1167T T1944C C1359T C669T T1239C
S. American fur seal/Argentina/RN-PB019/2023 ? T1944C C1359T C669T T1239C
S. American sea lion/Argentina/RN-PB004/2023 A1167T T1944C C1359T C669T T1239C
S. American tern/Argentina/CH-PD037/2023 A1167T T1944C C1359T C669T T1239C
Sea lion/Argentina/3893-1/2023 A1167T T1944C C1359T C669T T1239C
Sea lion/Argentina/RN-PB013/2023 A1167T T1944C C1359T C669T T1239C
S. American tern/Argentina/RN-PB015/2023 A1167T T1944C C1359T C669T T1239C
S. elephant seal/Argentina/CH-PD035/2023 A1167T T1944C C1359T C669T T1239C
S. American sea lion/Argentina/RN-PB011/2023 A1167T T1944C C1359T C669T C1002T T1239C

Supplementary Figure 10. Proximity between southern elephant seals and South American sea lions at Península Valdés, Argentina. Three adult southern elephant seals and their respective pups can be seen to the right of the image, in close proximity to a large number of South American sea lions (mostly adult females, with one adult male at the center top) to the left of the image. Photo taken at Punta Buenos Aires.

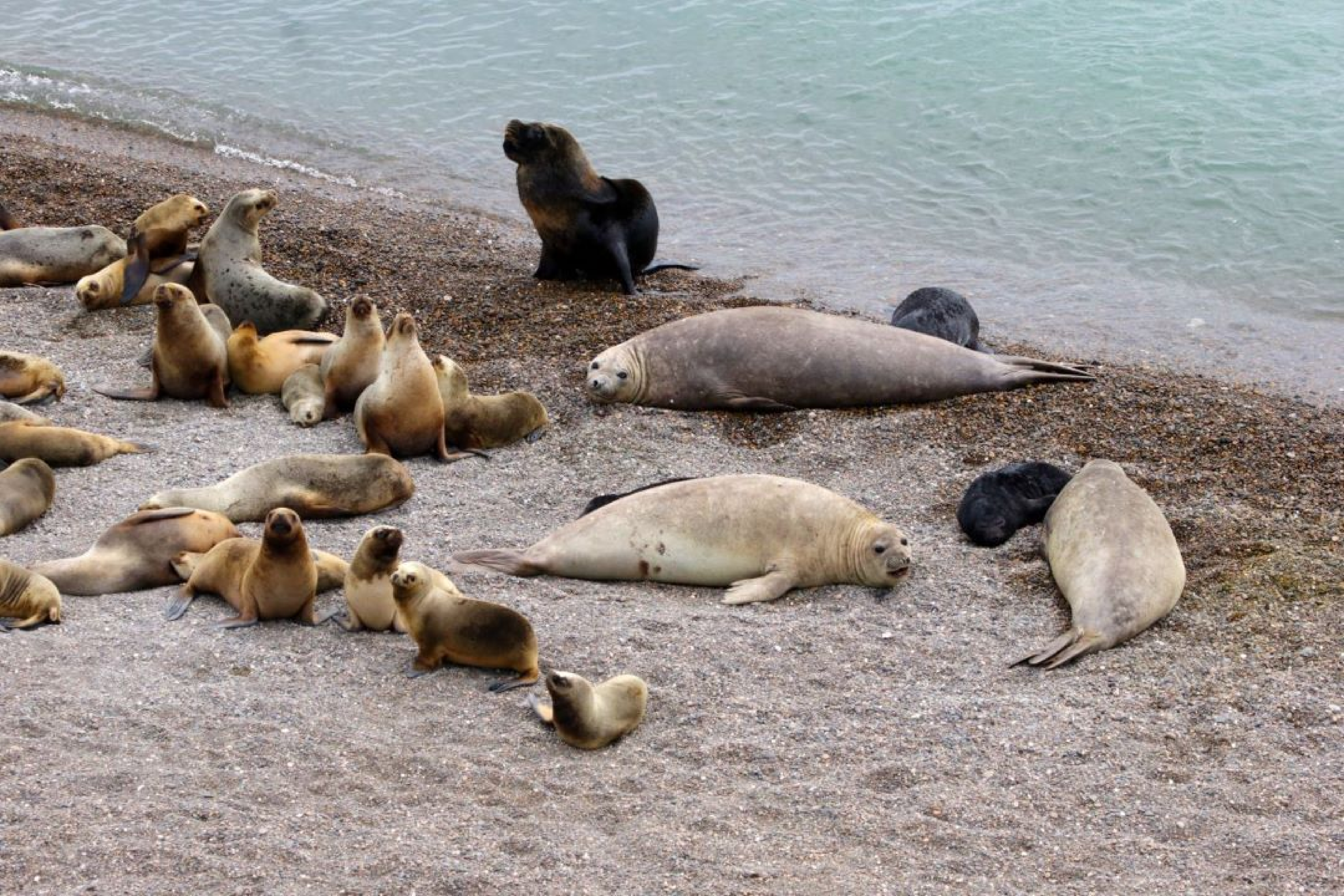

Supplement: Supplementary file 1 — Supplementary Information [file 41467_2024_53766_MOESM1_ESM.pdf]
