# Supplementary material for: Epidemiological data of an influenza A/H5N1 outbreak in elephant seals in Argentina indicates mammal-to-mammal transmission
Source: Nat Commun. 2024 Nov 11;15:9516. doi: 10.1038/s41467-024-53766-5 (PMC11555070; doi:10.1038/s41467-024-53766-5)
Supplement: Supplementary file 2 — Description of Additional Supplementary Files [file 41467_2024_53766_MOESM2_ESM.docx]

Description of Additional Supplementary Files

**File Name:** Supplementary Movie 1

**Description:** Elephant seals showing neurological and respiratory clinical signs during the 2023 outbreak of H5N1 HPAI in Península Valdés, Argentina.
