# Supplementary material for: Epidemiological data of an influenza A/H5N1 outbreak in elephant seals in Argentina indicates mammal-to-mammal transmission
Source: Nat Commun. 2024 Nov 11;15:9516. doi: 10.1038/s41467-024-53766-5 (PMC11555070; doi:10.1038/s41467-024-53766-5)
Supplement: Supplementary file 4 — Reporting Summary [file 41467_2024_53766_MOESM4_ESM.pdf]

nature portfolio

Corresponding author(s): Uhart, Marcela M and Rimondi, Agustina

Last updated by author(s): Oct 17, 2024

Reporting Summary

Nature Portfolio wishes to improve the reproducibility of the work that we publish. This form provides structure for consistency and transparency in reporting. For further information on Nature Portfolio policies, see our Editorial Policies and the Editorial Policy Checklist.

Statistics

For all statistical analyses, confirm that the following items are present in the figure legend, table legend, main text, or Methods section.

n/a	Confirmed	
☒	☐	The exact sample size (*n*) for each experimental group/condition, given as a discrete number and unit of measurement
☒	☐	A statement on whether measurements were taken from distinct samples or whether the same sample was measured repeatedly
☒	☐	The statistical test(s) used AND whether they are one- or two-sided<br>*Only common tests should be described solely by name; describe more complex techniques in the Methods section.*
☒	☐	A description of all covariates tested
☒	☐	A description of any assumptions or corrections, such as tests of normality and adjustment for multiple comparisons
☒	☐	A full description of the statistical parameters including central tendency (e.g. means) or other basic estimates (e.g. regression coefficient) AND variation (e.g. standard deviation) or associated estimates of uncertainty (e.g. confidence intervals)
☒	☐	For null hypothesis testing, the test statistic (e.g. *F*, *t*, *r*) with confidence intervals, effect sizes, degrees of freedom and *P* value noted<br>*Give P values as exact values whenever suitable.*
☐	☒	For Bayesian analysis, information on the choice of priors and Markov chain Monte Carlo settings
☒	☐	For hierarchical and complex designs, identification of the appropriate level for tests and full reporting of outcomes
☒	☐	Estimates of effect sizes (e.g. Cohen's *d*, Pearson's *r*), indicating how they were calculated

Our web collection on statistics for biologists contains articles on many of the points above.

Software and code

Policy information about availability of computer code

Data collection	no software was used for data collection
Data analysis	several software packages were used for data analysis and are presented throughout the methods section. These include: Maximum likelihood (ML) methods available in IQ-Tree 2; Biowulf Linux cluster at the National Institutes of Health (http://biowulf.nih.gov); MAFFT v7.490; CLC Genomics Workbench v23.0.2 (Qiagen); BEAST package pre-release v1.10.5 (compiled on 24-Apr-2023); BEAGLE 3 library; Tracer version 1.7.2; LogCombiner v1.10.4; TreeAnnotator v1.10.4; host-specific local clock (HSLC); R v4.3.2. All software is publicly available at https://github.com/beast-dev/beast-mcmc<br>https://github.com/jlcherry/SAMPI

For manuscripts utilizing custom algorithms or software that are central to the research but not yet described in published literature, software must be made available to editors and reviewers. We strongly encourage code deposition in a community repository (e.g. GitHub). See the Nature Portfolio guidelines for submitting code & software for further information.

Data

Policy information about availability of data

All manuscripts must include a data availability statement. This statement should provide the following information, where applicable:

- Accession codes, unique identifiers, or web links for publicly available datasets
- A description of any restrictions on data availability
- For clinical datasets or third party data, please ensure that the statement adheres to our policy

We have included a data availability statement: The sequence data generated in this study have been deposited in GenBank under accession codes PQ002111–PQ002158 and PP488310–PP488349. The extensible markup language (XML), maximum clade credibility (MCC), and maximum likelihood (ML) trees, host-specific local clock (HSLC) model files, Markov jump analysis files, GISAID acknowledgement tables, and underlying data for raw tree files are provided in Zenodo [https://doi.org/10.5281/zenodo.13923371]. Source data are provided with this paper.

Research involving human participants, their data, or biological material

Policy information about studies with human participants or human data. See also policy information about sex, gender (identity/presentation), and sexual orientation and race, ethnicity and racism.

Reporting on sex and gender	not applicable
Reporting on race, ethnicity, or other socially relevant groupings	not applicable
Population characteristics	not applicable
Recruitment	not applicable
Ethics oversight	not applicable

Note that full information on the approval of the study protocol must also be provided in the manuscript.

Field-specific reporting

Please select the one below that is the best fit for your research. If you are not sure, read the appropriate sections before making your selection.

☒ Life sciences ☐ Behavioural & social sciences ☐ Ecological, evolutionary & environmental sciences

For a reference copy of the document with all sections, see nature.com/documents/nr-reporting-summary-flat.pdf

Life sciences study design

All studies must disclose on these points even when the disclosure is negative.

Sample size	Sample size was limited to availability of freshly dead animals during avian influenza outbreak investigation, and included four elephant seal pups, one subadult male, six South American terns and two royal terns . All samples collected were tested to confirm avian influenza infection and elephant seal samples for H5 and clade confirmation. Eleven samples were selected for full genome sequencing. Details are provided in methods and results. Not applicable. The epidemiological component of the study is essentially descriptive, hence there were no covariates to be controlled for. The epidemiological component of the study is essentially descriptive, hence there were no covariates to be controlled for.
Data exclusions	No data were excluded, other than suboptimal quality reads in full genome sequencing and singleton marine mammal viruses positioned in the major avian clade, following host-specific local clock evolutionary analysis. The rationale for these are detailed in methods.
Replication	Diagnostics were confirmed by phylogenetic analysis. To assess robustness of each node in the tree, a bootstrap process with 1000 replicates was performed. Likewise, 500 bootstrap replicates were used for comparison of study findings with others from South America. All is described in methods.
Randomization	Samples were not collected randomly. They were selected from the most fresh carcasses found during the outbreak. For viral sequencing, lower Ct values were used to select best quality samples. All is detailed in methods and supplementary materials.
Blinding	Blinding was not relevant for this study. The study relies on samples collected from wildlife found dead with no prior knowledge on the cause of death. Carcasses were selected for sample collection based on the level of degradation (samples were only collected from carcasses in rigor mortis to ensure that the tissues were as fresh as possible).<br>At the laboratory, team members running the RT-qPCR tests had access to basic metadata (species, age group, site and date of sample collection) as this was necessary to determine sample pooling strategy, but without knowledge of additional information (e.g. ante-mortem clinical signs, necropsy findings) that could provide insight on whether a specific sample was more or less likely to be positive for HPAI. Nevertheless, this was irrelevant because all individuals tested ended up being positive for HPAI H5N1.

Reporting for specific materials, systems and methods

We require information from authors about some types of materials, experimental systems and methods used in many studies. Here, indicate whether each material, system or method listed is relevant to your study. If you are not sure if a list item applies to your research, read the appropriate section before selecting a response.

Materials & experimental systems

n/a	Involved in the study
☒	☐ Antibodies
☒	☐ Eukaryotic cell lines
☒	☐ Palaeontology and archaeology
☐	☒ Animals and other organisms
☒	☐ Clinical data
☒	☐ Dual use research of concern
☒	☐ Plants

Methods

n/a	Involved in the study
☒	☐ ChIP-seq
☒	☐ Flow cytometry
☒	☐ MRI-based neuroimaging

Animals and other research organisms

Policy information about studies involving animals; ARRIVE guidelines recommended for reporting animal research, and Sex and Gender in Research

Laboratory animals	the study did not involve laboratory animals
Wild animals	the study only involved dead wild animals.
Reporting on sex	Sex of sampled animals was recorded but was not part of the study design since samples were collected opportunistically from the freshest carcasses available and aiming for a representative number of individuals from affected species.
Field-collected samples	Laboratory work from field collected samples was limited to diagnostic confirmation of influenza A infections and subsequent molecular and genetic characterization of viruses.
Ethics oversight	Ethical committee approval was not required since the study solely focused on sample collection from animals found dead. No samples were collected from living animals.

Note that full information on the approval of the study protocol must also be provided in the manuscript.

Plants

Seed stocks	not applicable
Novel plant genotypes	not applicablenot applicable
Authentication	not applicable
